# Energetic considerations for engineering novel biochemistries in photosynthetic organisms

**DOI:** 10.3389/fpls.2023.1116812

**Published:** 2023-02-06

**Authors:** Deserah D. Strand, Berkley J. Walker

**Affiliations:** ^1^ U. S. Department of Energy (DOE) Plant Research Laboratory, Michigan State University, East Lansing, MI, United States; ^2^ Department of Plant Biology, Michigan State University, East Lansing, MI, United States

**Keywords:** photosynthesis, chloroplast bioenergetics, synthetic biology, chloroplast biotechnology, bioenergetics

## Abstract

Humans have been harnessing biology to make valuable compounds for generations. From beer and biofuels to pharmaceuticals, biology provides an efficient alternative to industrial processes. With the continuing advancement of molecular tools to genetically modify organisms, biotechnology is poised to solve urgent global problems related to environment, increasing population, and public health. However, the light dependent reactions of photosynthesis are constrained to produce a fixed stoichiometry of ATP and reducing equivalents that may not match the newly introduced synthetic metabolism, leading to inefficiency or damage. While photosynthetic organisms have evolved several ways to modify the ATP/NADPH output from their thylakoid electron transport chain, it is unknown if the native energy balancing mechanisms grant enough flexibility to match the demands of the synthetic metabolism. In this review we discuss the role of photosynthesis in the biotech industry, and the energetic considerations of using photosynthesis to power synthetic biology.

## Introduction

Plants and algae are attractive platforms for bioengineering pathways for valuable metabolic products because they use freely available solar energy to sustainably power synthetic biology. To make best use of this energy, the localization of engineered synthetic metabolism should be carefully considered relative to where the metabolic precursors are produced. In the cytosol and other non-energetic subcellular compartments, the metabolic precursors ATP and reducing equivalents (e.g., NADPH and ferredoxin) required for synthetic metabolism must be supplied by other metabolism or transported from the energetic compartments (i.e., mitochondria or chloroplast). For synthetic metabolism with large demands of ATP and/or reducing equivalents, localization to the chloroplast allows the synthetic metabolism direct access to ATP and reducing equivalents generated by the light dependent reactions in the chloroplast. Traditional bioengineering approaches usually focus on engineering carbon flux but seldom think about the types of energy fluxes required to power synthetic metabolism. In point of fact, one should not consider the light reactions as an independent supplier of ATP or NADPH because their production is intrinsically linked. The light dependent reactions in the thylakoid electron transport chain are constrained to produce a fixed stoichiometry of ATP and reducing equivalents that may not match the newly introduced synthetic metabolism, leading to inefficiency or damage. While photosynthetic organisms have evolved several ways to modify the ATP/NADPH output from their thylakoid electron transport chain, it is unknown if the native energy balancing mechanisms grant enough flexibility to match the demands of the synthetic metabolism. In other words, is native photosynthetic energy production able to support maximal yields of bioproduction from engineered photosynthetic organisms, or will photosynthesis itself need to be re-engineered?

In this review, we discuss bioengineering from an energetics perspective and where this may present a hurdle for using photosynthetic organisms for producing bioproducts of interest.

## The chloroplast as a factory

The chloroplast sets photosynthetic eukaryotes apart from other organisms for bioengineering. The chloroplast is the solar cell of the plant, utilizing light energy to generate ATP and reducing power for use in downstream metabolism. This makes these organisms attractive as a bioengineering platform for synthetic metabolism for valuable products because growth and yield is sustained by light; a cheap, abundantly available natural resource. Additionally, the chloroplast itself is the site of many metabolic precursors that can be harnessed for synthetic metabolism (e.g., ATP, reducing equivalents, sugar phosphates, fatty acids, isoprenoids, and amino acids). Enzymes in a desired synthetic pathway that utilize these precursors may be expressed in the nucleus of eukaryotes and targeted to the chloroplast, or integrated into the chloroplast genome, with both approaches having their place in bioengineering.

There are tradeoffs that must be considered when choosing to express transgenes from either the nucleus or the chloroplast. Most nuclear transformation techniques result in random transgene integration and a variety of transgene expression levels. Chloroplast transformation, however, allows for directed integration of transgenes and every chloroplast transformant is genetically identical, resulting in reproducible expression (Bock, 2001; Bock, 2015; Ahmad et al., 2016). Nuclear transformation may lead to undesirable transgene silencing in later generations (De Wilde et al., 2000). While there are approaches to avoid silencing and increase transgene expression in the nucleus, chloroplast transgenes are not subject to silencing, resulting in continued high expression generations later. Therefore, chloroplast transformation may be an approach to avoid the silencing problem completely if the transgene product is ultimately desired in the chloroplast (Bock, 2001).

There are vastly more species with transformable nuclei than chloroplasts, but the number of valuable species amenable to chloroplast transformation is constantly increasing, and established protocols exist for many model organisms and economically important crops (e.g., soybean) (Boynton et al., 1988; Svab and Maliga, 1993; Ahmad et al., 2016; Nimmo et al., 2019; Kaushal et al., 2020; Ruf et al., 2021). Additionally, the molecular toolbox for control of chloroplast transgene expression is continually growing, offering synthetic biologists a variety of options to fine-tune expression levels and timing (Bock, 2022). These molecular transformation tools allow us to utilize the chloroplast as a molecular factory, using the raw materials of the chloroplast to supply the production of valuable molecules. However, synthetic metabolism will be in competition for energy and carbon flux within the native chloroplast metabolism. Strategies for optimizing efficiency and yield of our synthetic metabolism will need to account for the demands of the chloroplast’s native metabolism, and how those demands are currently supplied.

## The native chloroplast energy demand

Natively, the largest sinks for the ATP and reducing equivalents in the chloroplast are the Calvin-Benson-Bassham (CBB) cycle and photorespiration (Noctor and Foyer, 1998; Walker et al., 2020). The CBB cycle begins when ribulose 1,5 bisphosphate carboxylase/oxygenase (rubisco) catalyzes reaction with CO_2_ (carboxylation reaction) and ribulose 1,5 bisphosphate (RuBP), forming two 3-carbon molecules of 3-phosphoglycerate (PGA). PGA is converted to 1,3-bisphosphoglycerate and then to glyceraldehyde-3-phosphate, a portion of which is used in the construction of glucose and other sugars with the remainder feeding back into the regeneration of RuBP, consuming ATP and NADPH in a stoichiometry of 3:2. Alternatively, when rubisco catalyzes reaction with O_2_ (oxygenase reaction), one molecule of PGA and one molecule of 2-phosphoglycolate (PG) are formed (Ogren, 1984; Foyer et al., 2009). The PG is inhibitory and must be detoxified in a series of enzymatic reactions that occur across three organelles in a process termed photorespiration (PR) which loses CO_2_ in the process. Energetically, the whole-cell demand ratio for ATP : NADPH for photorespiration is 1.75 (Edwards and Walker, 2003; Walker et al., 2020). Regardless of the assumptions used for determining the whole-cell energy demand of photorespiration, the conversion of glycerate to PGA requires an ATP within the chloroplast. This increased consumption in the chloroplast means that in the absence of ATP or NADPH transport, photorespiration increases the relative ATP demand within the chloroplast above that of the CBB cycle.

Other pathways in the chloroplast have higher relative reductant demands than the CBB cycle and PR. Both synthesis of fatty acids and desaturation of lipids require reducing equivalents in the form of NADPH, making the demand for reducing equivalents from lipid biosynthesis larger that of the CBB cycle (Ohlrogge and Browse, 1995). Additionally, the methylerythritol 4-phosphate portion of the isoprenoid biosynthesis pathway in the chloroplast also has a larger demand for reducing equivalents relative to ATP (Zhao et al., 2013). While under normal conditions these metabolic fluxes are much smaller than that of the CBB cycle and PR, metabolic engineering with the goal of increasing flux through these pathways would increase the relative reductant demand within the chloroplast.

## The chloroplast energy supply

Since the light reactions of photosynthesis provide all the energy for native and synthetic metabolism in autotrophs, it is essential to understand how light energy is converted to usable chemical energy and what constrains production of this chemical energy. It is also important to understand the constraints to this energy production for optimal bioengineering of reliant synthetic metabolism.

**Figure 1:**
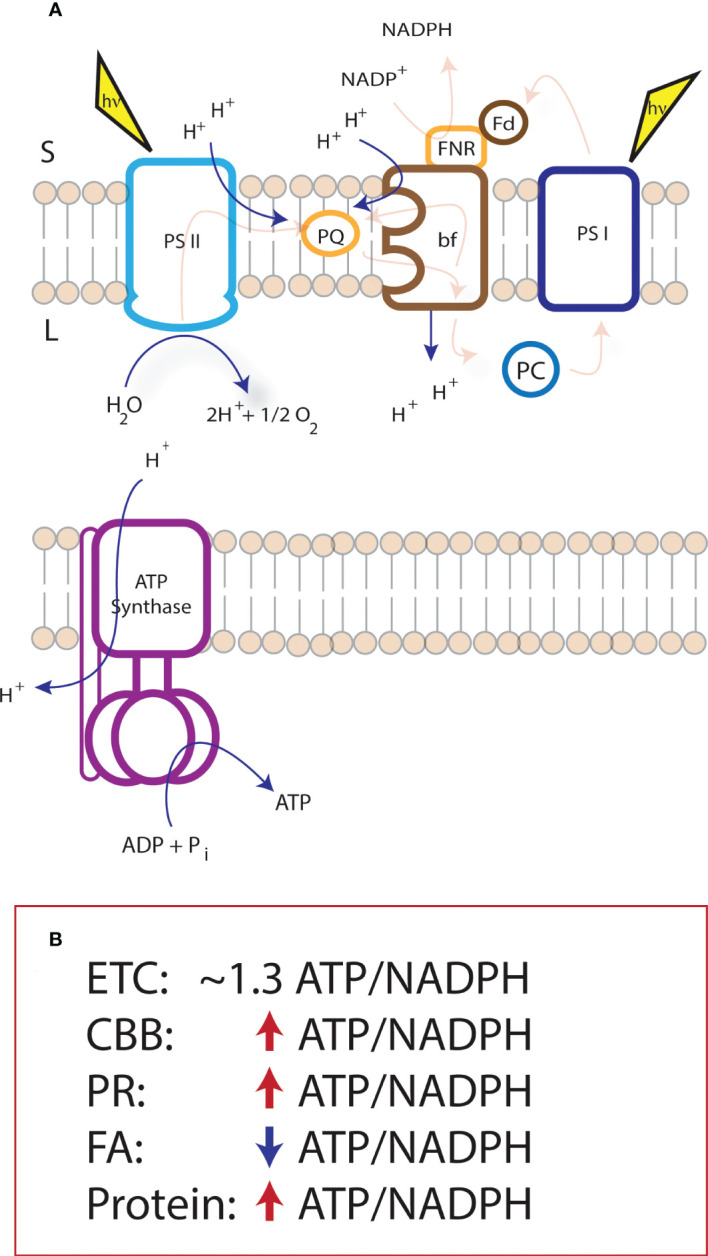
Supply and demand of NADPH and ATP within the chloroplast. The proton to electron stoichiometry of proton coupled electron transfer of the light reactions of photosynthesis (as pictured in panel **A**) is fixed at 3H^+^/e^-^ and, together with the c-ring structure of the ATP synthase, constrains the electron transport chain production to ~1.3 ATP/NADPH. The light reactions supply multiple metabolic sinks within the chloroplast that do not match the ATP/NADPH resulting in an ATP deficit or surplus. **(B)** indicates pathways of interest for metabolic engineering, and their difference in demand (within the chloroplast) from the ATP/NADPH production of the electron transport chain. CBB, Calvin-Benson-Bassham cycle; PR, photorespiration; FA, Fatty acid biosynthesis; Protein, Protein synthesis.

The light reactions of photosynthesis use light excitation to drive a series of redox reactions along an electron transport chain (ETC) to shuttle protons and electrons across the thylakoid membrane generating ATP and reducing power for downstream metabolism[**Figure 1**, (Hill and Bendall, 1960; Arnon, 1971; Mitchell, 1975; Blankenship, 2002; Mitchell, 2011)]. The coupling of these proton and electron circuits determine the ATP/NADPH produced from the system. When light hits the photosystems, the excited reaction centers lose an electron. At photosystem II (PSII), electron loss at P680 supplies the oxidative force (P680^+^) to release electrons from water, reducing P680^+^ and releasing protons into the lumen. Electron transfer falls downhill in energy to plastoquinone (PQ), the reduction of which leads to the uptake of protons from the stroma. These protons are released into the lumen when plastoquinol (PQH_2_) is oxidized at the cytochrome *bf* (cyt *bf*) complex. Electron transfer continues through plastocyanin to reduce the oxidized photosystem I (PSI) reaction center, P700^+^. When light energy oxidizes P700, the electron travels through PSI to the soluble electron carrier ferredoxin which can provide reducing power to metabolism, redox regulation, or be used to reduce NADP^+^ to NADPH. NADPH is used as a two-electron donor to a wide variety of metabolism in the chloroplast as discussed above.

The proton-coupled electron transfer reactions at PSII and cyt *bf* lead to proton accumulation in the thylakoid lumen, generating an electrochemical proton gradient used for ATP synthesis at the ATP synthase. At PSII, water splitting results in the release of one proton into the lumen per electron entering the electron transport chain. When PQH_2_ is oxidized at the cyt *bf* complex, electrons enter a bifurcated pathway, one of which is eventually passed to PSI, and another is passed to another site on cyt *bf* to half reduce another PQ [i.e., the Q-cycle, (Mitchell, 1975)]. Therefore, for each electron passed from cyt *bf* to PSI, two protons are deposited into the lumen. Across the membrane, the difference in charge imposed by this proton gradient comprises a membrane potential (Δψ) and the difference in pH generates a pH gradient (ΔpH) (Cruz et al., 2001). Together, these comprise the protonmotive force (Δ*p*) for ATP synthesis *via* the ATP synthase (Nicholls and Ferguson, 2002; Kramer et al., 2003). The chloroplast ATP synthase is comprised of two complexes, the CF_o_ in the membrane, and the CF_1_ in the stroma (Hahn et al., 2018). The CF_o_ contains a 14-subunit c-ring and a proton half channel, releasing 14 protons per one full turn (Seelert et al., 2003). This turn moves the g-subunit, which in turn drives the conformational change in the CF_1_ domain, comprised of 3 heterodimers, and responsible for ATP synthesis. Therefore, 14 protons are required to synthesize 3 ATP, and NADP^+^ reduction to NADPH requires 2 electrons from the electron transport chain.

From the H^+^/e^-^ at PSII and cyt *bf*, there is a total stoichiometry of the ETC of 3H^+^/e^-^, which results in an ATP/NADPH of ~1.3, in a perfectly coupled system. This means that, the largest sinks for ATP and NADPH in the chloroplast (i.e., CBB cycle and photorespiration) are running at an ATP deficit. The ATP/NADPH production stoichiometry is flexible to a certain extent by the activation of, or leak of electrons into, alternative electron transfer pathways [discussed in depth elsewhere, e.g., (Kramer and Evans, 2011; Strand et al., 2016; Alric and Johnson, 2017; Walker et al., 2020)]. The most studied alternative pathways lead to an increase in the relative production of ATP from the ETC. This is likely due to the largest sinks in the chloroplast imposing an ATP deficit on the ETC (discussed below), however, with the introduction of synthetic biology into the chloroplast, we must consider the impact of new or altered metabolic sinks on chloroplast energetics.

## Consequences of energetic imbalance

To optimize efficiency and avoid damage, the chloroplast must balance the supply and demand of ATP and reducing equivalents. Energy demand from native metabolism in the chloroplast does not match the calculated output of the light reactions, therefore it is likely that throughout the organism’s life cycle it experiences periods of ATP deficits or surplus. What problem do these conditions pose for the plant?

**Figure 2:**
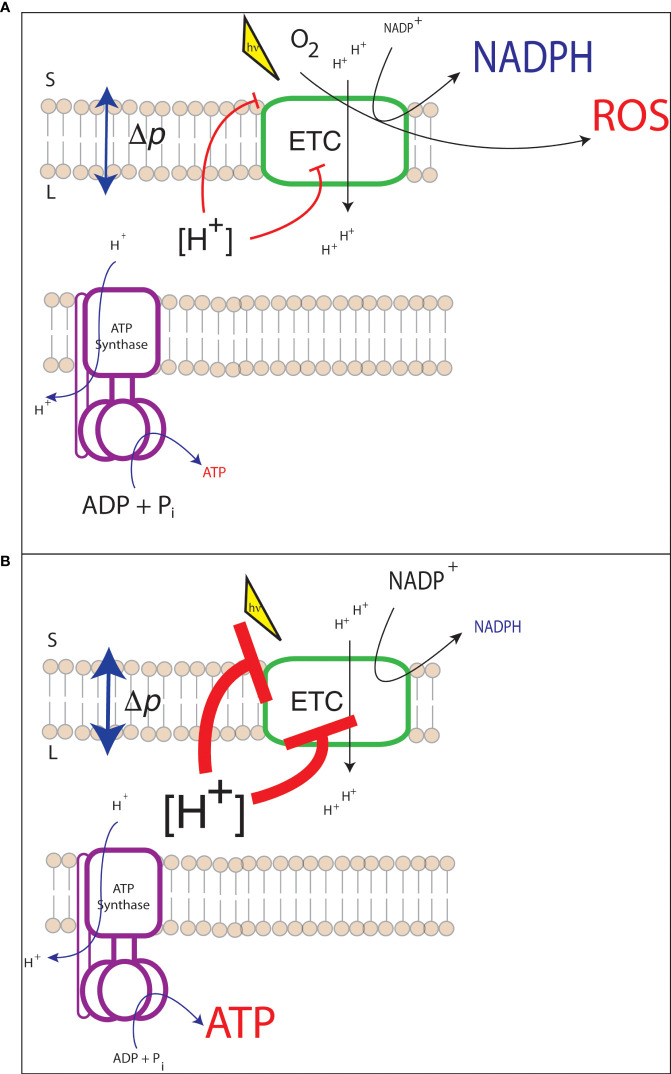
Possible consequences of energetic mismatch between the electron transport chain (ETC) and downstream metabolism. **(A)** An ATP deficit may result in the closure of the acceptor side of PSI and the generation of harmful reactive oxygen species (ROS); **(B)** An ATP surplus may lead to substrate limitation of ATP synthase and the downregulation of light harvesting and electron transfer resulting in lower photosynthetic efficiency.

When ATP demand relative to NADPH exceeds the supply of the light reactions (ATP deficit, **Figure 2A**), sinks for reducing power may be closed as they do not have ATP as substrate to turnover acceptors. For example, if ATP is limiting to the CBB cycle, 1,3-bisphosphoglycerate is not produced from 3-phosphoglycerate. Since 1,3-bisphosphoglycerate is an electron acceptor, NADP^+^ is not being regenerated for reduction by PSI. This shifts the NADPH/NADP^+^ to a more reduced state, lowering the redox potential of the acceptor side of PSI. A more reduced acceptor side of PSI is dangerous because it will increase the likelihood of electrons being passed to oxygen. Reduction of oxygen generates superoxide ( ${\text{O}}_{2}^{.-}$ ), a reactive oxygen species that can damage protein and lipid, and must be detoxified [i.e., through the water-water cycle, (Asada, 2000)]. This detoxification itself diverts electrons from NADPH, and activates another electron sink (i.e, cyclic electron flow) to increase ATP/NADPH production (Casano et al., 2001; Strand et al., 2015).

However, if a large enough ATP sink was introduced into the chloroplast, it is not clear if the self-balancing machinery of the chloroplast is robust enough to meet the new demand without side effects like decreased growth. An example of when the self-balancing machinery fails to meet a new metabolic demand may be seen in a mutant of the chloroplast fructose 1,6 bisphosphatase (FBPase) (Livingston et al., 2010). This mutant seemingly survives disruption to the main metabolic sink in the chloroplast by bypassing the FBPase lesion though the glucose-6-phosphate shunt, which has increased relative ATP demand (Sharkey and Weise, 2016). Despite activation of multiple alternative electron transfer pathways to increase ATP supply from the ETC (Livingston et al., 2010; Strand et al., 2015), these plants grow slower and smaller.

This may, in part, be due to the secondary impact cyclic electron flow around PSI (CEF), an alternative electron transport pathway that is increased in the FBPase knockout which may decrease the efficiency of photosynthesis. CEF increases proton translocation into the lumen relative to electron transfer from PSII. Additional proton translocation can increase the Δ*p* in the lumen, activating pH dependent feedback regulation i.e., downregulation of light harvesting (exciton quenching, q_E_) and electron transfer at cyt *bf* (photosynthetic control, PCON). In other words, the responses used to mitigate the ATP deficit may lead to total decrease in output of the light reactions. In an industrial process, this would be an undesired side effect limiting the yield of the engineered pathways.

While the chloroplast has many identified routes of alternative electron transfer to deal with an ATP deficit, much less in known about how an ATP surplus (or, alternatively, a reducing equivalent deficit). For those routes that are proposed, there is little evidence that they contribute substantially to chloroplast energy balancing in the light (Flügge et al., 2011; Strand et al., 2017). The prevalence of pathways to mitigate an ATP deficit rather than mitigate an ATP surplus may be due to selective pressure stemming from native metabolism being poised towards an ATP deficit as discussed above (Walker et al., 2020). However, many proposed synthetic biology pathways result in an ATP surplus such as increased lipid production in microalgae and photorespiratory bypasses in plants (Miller et al., 2020).

Under an ATP surplus condition the threat of damage to the chloroplast is not a primary concern, instead the efficiency of the light reactions will decrease. This inefficiency would occur through a shift in the [ATP]/[ADP] [P_i_] pool which may lead to an increase in Δ*p* required to drive ATP synthesis. All else being equal, increased Δ*p* leads to an activation of q_E_ and PCON (**Figure 2B**), decreasing light harvesting and electron transfer rates. In the case of a synthetic pathway with an increased electron demand, activation of these photoprotective processes would lead to decreased yield of the synthetic pathway. This response to increased electron demand may be a limiting factor for any synthetic pathway with high reductant demand, such as increased lipid production.

The conditions described above are an important consideration for bioengineering pathways relying on output from the light reactions of photosynthesis. This is because final product yield may ultimately be limited by the light reactions if new metabolism exceeds the innate energetic flexibility of the chloroplast, even if the engineered metabolism yields the expected product to some degree. To increase product yield, it may be enticing to modify the regulatory processes that are activated in response to energetic imbalance. However, feedback regulation that limits light utilization and electron transfer (i.e., q_E_ and PCON) are important to protect the photosystems from photodamage and in the case of PCON, it is *essential* for plant survival under fluctuating conditions (Müller et al., 2001; Suorsa et al., 2012). This is an important point: energetic limitations can’t simply be rectified by eliminating feedback regulation, and instead may require additional engineering to optimize our desired yield.

## Case studies in chloroplast energetics

Since the invention of plant transformation, there have been many successful applications of bioengineering metabolism to produce valuable metabolites, either by the introduction of synthetic metabolism or altering the flux through metabolic pathways to favor a specific molecule [reviewed in detail in (O’Neill and Kelly, 2017)]. While there have been many successes without energetic considerations, it may be worthwhile to consider the ATP and NADPH requirements when yield from synthetic pathways is lower than expected. Here we offer examples of synthetic metabolism that, if successfully implemented into a photosynthetic organism would drastically alter the energetic demands in the chloroplast and may need additional engineering to ensure maximal yield of the desired product.

Due to the loss of CO_2_ during the detoxification and recycling of PG, photorespiration is thought to be a large inefficiency in C3 plants and is an active area of research for improvement of crop productivity (Fu et al., 2022b). There have been several approaches to decrease the rate of photorespiration, e.g., by introducing a bypass modifying the compartment of CO_2_ release (Peterhansel et al., 2010; South et al., 2019), or compartmentalization of rubisco (Lin et al., 2014). However, an alternative approach to decreasing biomass loss due to photorespiration is the complete bypass of rubisco to fix carbon. One approach that has been shown to operate *in vitro* is the so called ‘Cetch’ cycle (Schwander et al., 2016; Miller et al., 2020), a completely synthetic pathway for CO_2_ fixation achieved by combining enzymes from multiple organisms across the tree of life. This approach is interesting due the pathway’s increased relative demand in reducing equivalents. The challenges of introducing this cycle into a plant are extensive (e.g., expression, availability of cofactors), but the prospect does introduce an intriguing case study of the need for multiple engineering targets to optimize yield. Since the demand of this cycle for reductant is extremely altered (0.66 ATP/NADPH) from that of native CO_2_ fixation (1.5 ATP/NADPH), organisms utilizing the ‘Cetch’ cycle would certainly require secondary bioengineering to overcome the substantial change in chloroplast energetics for growth to be sustainable.

Another engineering target is in the use of photosynthetic organisms to produce biofuel feedstocks (Blatti et al., 2013). One attractive approach to sustainable biofuel production is the use of lipid rich algae. There has been substantial research effort into both identifying algal species that are suitable for robust lipid production and developing molecular toolkits to allow for their genetic modification (Alishah Aratboni et al., 2019). While there have been some successes in increasing lipid production or shifting lipid production to more valuable lipid species, more effort is needed to make algae an economically viable biofuel feedstock (Blatti et al., 2013; Alishah Aratboni et al., 2019; Gilmour, 2019; Verma and Kuila, 2020). From an energetic (i.e., ATP : NADPH budget) perspective, increasing relative lipid production has different considerations than increasing overall biomass. The latter may be approached by looking at metabolic bottlenecks, but substantial flux through the fatty acid biosynthetic pathway, with its relatively high NADPH demand, may introduce a bottleneck to the light reactions (**Figure 2B**).

Due to the targeted nature of synthetic biology, the energetic demands of the synthetic pathway can be determined from its ATP and reductant demands. This presents an opportunity to incorporate the new metabolism into existing models [e.g., (Noctor and Foyer, 1998)] and estimate the impact the synthetic pathways may have on cellular energetics. Several flux models exist quantifying metabolic flux under various environmental conditions (Ma et al., 2014; Wieloch and Sharkey, 2022; Fu et al., 2022a) and may help to gauge the impact introduction of new metabolic sinks may have on energetics relative to native metabolism. This could also serve as a starting point for the design of accessory metabolism to offset any energetic imbalances imposed by the introduced metabolism.

## Translation to crops

Broadly, increasing photosynthetic output by targeting inefficiencies has been shown to increase overall plant biomass [e.g.(Driever et al., 2017; South et al., 2019; Chen et al., 2020; Wang et al., 2020)]. In biofuel crops where general biomass is utilized for fermentation, this will likely result in the increased yield of desired product, but most bioengineering approaches are concerned with increasing a specific plant product (e.g., grain, lipids). Due to the complex regulation of carbon partitioning in crops (Yu et al., 2015; Paul, 2021), it is unclear if targeting photosynthetic inefficiencies or energetics will translate to increased grain or fruit production.

Despite these potential issues, engineering approaches that increase CO_2_ assimilation have yielded higher overall biomass, including an increase in grain (Driever et al., 2017). Since many breeding successes have been measured by increasing yield while decreasing overall plant size [i.e., increased harvest index (HAY, 1995)], increasing grain yield by increasing overall crop biomass may increase the land use need, offsetting our perceived increases.

## Improving photosynthesis for growth and resilience

Despite the uncertainty of downstream partitioning of biomass, photosynthesis researchers have a goal focused on improving the efficiency of photosynthesis (Long et al., 2006; Ort et al., 2015). With increased understanding of the regulation of these processes, we may increase the amount of light energy harnessed into driving CO_2_ assimilation. Regardless of the downstream success in biomass partitioning, these increases will be an important building block in our steps to improve crops. There have been several approaches taken to improving the electron transport chain, many of which have resulted in small increases photosynthetic efficiency and/or in downstream carbon assimilation or overall plant biomass.

One large target of improvement is the group of processes activated by the accumulation of the ΔpH component of Δ*p* (i.e., q^E^ and PCON). Even though q_E_ and PCON are important to protect the thylakoid electron transport chain from damage, they are often cited as points for crop improvement to minimize energy loss and increase electron transfer rates (Murchie and Niyogi, 2011). In the case of an ATP surplus introduced by synthetic biology, these processes may also be a target to increase electron transfer to synthetic electron sinks. This should be undertaken with caution, as it may seem attractive to alter the sensitivity of these photoprotective processes to increase electron transfer, but this approach will also open our organisms to increased risk of photodamage. Under controlled environmental conditions, this may not be problematic, however, if engineered plants are grown in greenhouse or field conditions, they will experience rapidly changing light conditions. Without feedback regulation excess excitation energy can lead to the damage of PSII which must be repaired, leading to the long-term downregulation of light harvesting (Müller et al., 2001). Additionally, under high or rapidly fluctuating light, unregulated transfer of electrons through cyt *bf* can lead to damage of PSI, a potentially lethal situation for the plant (Suorsa et al., 2012).

Despite these concerns, there has been limited success with approaches to increase the flexibility of feedback regulation. Overexpression of cyt *bf* components (the rate limiting step of the electron transport chain) in a C4 plant has led to small increases in electron transfer and CO_2_ assimilation (Ermakova et al., 2019). This suggests there is some limitation due to protein content of the cyt *bf* complex, and that subtle increases of electron transfer through *bf* may be a route to improving photosynthetic efficiency. This is especially of interest because overexpression of cyt *bf* would not impact dynamic PCON, maintaining PSI photoprotection. Increased expression of a K^+^/H^+^ antiporter involved in exchanging ΔpH for Δψ during fluctuating light changes increased photosynthetic efficiency due to the increased capacity to relax q_E_ (Armbruster et al., 2016). Overexpression of q_E_ components, which has been shown to alter the sensitivity of q_E_ to the ΔpH component of Δ*p* (Li et al., 2002), has led to biomass increases in tobacco and soybean (Kromdijk et al., 2016; De Souza et al., 2022). However, in Arabidopsis the results were opposite (Garcia-Molina and Leister, 2020). Interpretation of these conflicting results is complicated, as the xanthophyll cycle component of NPQ is intrinsically linked to abscisic acid biosynthesis, the plant hormone involved in plant water relations (Niyogi et al., 1998).

While the light reactions supply ATP and NADPH for downstream carbon assimilation, except for low light, they do not appear to be the major limitation on bulk photosynthetic yield. There have also been improvements in assimilation and growth increases when the carbon reactions of photosynthesis are targeted, without modification of the light reactions. For example, overexpression of sebdoheptulose 1,7 bisphosphatase, the rate limiting step in the CBB cycle leads to larger plants (Miyagawa et al., 2001; Driever et al., 2017), suggesting the bulk supply of the light reactions are able to meet increased demand from the carbon side. However, if we think about the system dynamically, there are parts of the day that plants are light limited, such as early morning. Under very low light, q_E_ and PCON are not active, and suppression of them wouldn’t likely offer substantial benefit. However, these processes are activated at intermediate light, where there is room to modify feedback regulation to improve photosynthesis (Murchie and Niyogi, 2011). Rather than modify the response of q_E_ and PCON directly, one approach that has potential is the modification of the metabolic sensor of the ETC, the ATP synthase.

The ATP synthase is the central regulator of photosynthesis. It senses downstream metabolic states and controls D*p* accumulation. For example, when the CBB cycle is substrate limited, the rate constant of proton efflux decreases, leading to the activation of q_E_ and PCON, even at low light (Kanazawa and Kramer, 2002). The ATP synthase is post-translationally regulated, the most understood of which is the light to dark inactivation of the redox regulated g- subunit (Kramer et al., 1990; Evron et al., 2000; Wu et al., 2007). However, the ATP synthase has also been proposed to be regulated by phosphorylation and protein-protein interactions, but these are not clearly understood (Evron et al., 2000; Bunney et al., 2001; Reiland et al., 2009). Additionally, it has been demonstrated that up to 50% of the ATP synthase content in tobacco leaves is inactive, and the molecular mechanism of this inactivation is completely unknown (Rott et al., 2011). Clearly, more basic research into the regulation of ATP synthase could lead to strategies to delay the onset of feedback regulation leading to q_E_ and PCON, and increase the integrated efficiency of the light reactions during the course of the day.

While the energetics of target pathways are not the only engineering consideration, we hope we have emphasized the importance of chloroplast energetics in synthetic biology. While some bioengineering strategies are promising, relatively extreme differences from native chloroplast metabolic demands may require secondary engineering to maximize the yield of the desired product. Finally, bioengineering of plants to fine tune energetic responses may lead to improved growth and resilience to stress conditions. Fine tuning should be considered as a strategy for plant improvement in addition to methods that may increase yield more dramatically.

## Author contributions

Both authors listed have made a substantial, direct, and intellectual contribution to the work and approved it for publication.

## References

[B1] Ahmad N. Michoux F. Lössl A. G. Nixon P. J. (2016). Challenges and perspectives in commercializing plastid transformation technology. J. Exp. Bot. 67, 5945–5960. doi: 10.1093/jxb/erw360 27697788

[B2] Alishah Aratboni H. Rafiei N. Garcia-Granados R. Alemzadeh A. Morones-Ramírez J. R. (2019). Biomass and lipid induction strategies in microalgae for biofuel production and other applications. Microb. Cell Fact. 18, 1–17. doi: 10.1186/s12934-019-1228-4 31638987 PMC6805540

[B3] Alric J. Johnson X. (2017). Alternative electron transport pathways in photosynthesis: a confluence of regulation. Curr. Opin. Plant Biol. 37, 78–86. doi: 10.1016/j.pbi.2017.03.014 28426976

[B4] Armbruster U. Leonelli L. Correa Galvis V. Strand D. Quinn E. H. Jonikas M. C.. (2016). Regulation and levels of the thylakoid K+/H+ antiporter KEA3 shape the dynamic response of photosynthesis in fluctuating light. Plant Cell Physiol. 57, 1557–1567. doi: 10.1093/pcp/pcw085 27335350 PMC4937787

[B5] Arnon D. I. (1971). The light reactions of photosynthesis. Proc. Natl. Acad. Sci. U. S. A. 68, 2883–2892. doi: 10.1016/1011-1344(92)87001-P 4400251 PMC389550

[B6] Asada K. (2000). The water-water cycle as alternative photon and electron sinks. Philos. Trans. R. Soc Lond. B. Biol. Sci. 355, 1419–1431. doi: 10.1098/rstb.2000.0703 11127996 PMC1692883

[B7] Blankenship R. (2002). Molecular mechanisms of photosynthesis. Ed. Blankenship R. E. (Oxford, UK: Blackwell Science Ltd). doi: 10.1002/9780470758472

[B8] Blatti J. L. Michaud J. Burkart M. D. (2013). Engineering fatty acid biosynthesis in microalgae for sustainable biodiesel. Curr. Opin. Chem. Biol. 17, 496–505. doi: 10.1016/j.cbpa.2013.04.007 23683348

[B9] Bock R. (2001). Transgenic plastids in basic research and plant biotechnology. J. Mol. Biol. 312, 425–438. doi: 10.1006/jmbi.2001.4960 11563907

[B10] Bock R. (2015). Engineering plastid genomes: Methods, tools, and applications in basic research and biotechnology. Annu. Rev. Plant Biol. 66, 211–241. doi: 10.1146/annurev-arplant-050213-040212 25494465

[B11] Bock R. (2022). Transplastomic approaches for metabolic engineering. Curr. Opin. Plant Biol. 66, 102185. doi: 10.1016/j.pbi.2022.102185 35183927

[B12] Boynton J. E. Gillham N. W. Harris E. H. Hosler J. P. Johnson A. M. Jones A. R.. (1988). Chloroplast transformation in chlamydomonas with high velocity microprojectiles. Science 240, 1534–1538. doi: 10.1126/science.2897716 2897716

[B13] Bunney T. D. Van Walraven H. S. De Boer A. H. (2001). 14-3-3 protein is a regulator of the mitochondrial and chloroplast ATP synthase. Proc. Natl. Acad. Sci. U. S. A. 9, 4249–4254. doi: 10.1073/pnas.061437498 PMC31211 11274449

[B14] Casano L. M. Martín M. Sabater B. (2001). Hydrogen peroxide mediates the induction of chloroplastic ndh complex under photooxidative stress in barley. Plant Physiol. 125, 1450–1458. doi: 10.1104/pp.125.3.1450 11244124 PMC65623

[B15] Chen J. H. Chen S. T. He N. Y. Wang Q. L. Zhao Y. Gao W.. (2020). Nuclear-encoded synthesis of the D1 subunit of photosystem II increases photosynthetic efficiency and crop yield. Nat. Plants 6, 570–580. doi: 10.1038/s41477-020-0629-z 32313138

[B16] Cruz J. A. Sacksteder C. A. Kanazawa A. Kramer D. M. (2001). Contribution of electric field (Delta psi) to steady-state transthylakoid proton motive force (pmf) *in vitro* and *in vivo.* control of pmf parsing into delta psi and delta pH by ionic strength. Biochemistry 40, 1226–1237. doi: 10.1021/bi0018741 11170448

[B17] De Souza A. P. Burgess S. J. Doran L. Hansen J. Manukyan L. Maryn N.. (2022). Soybean photosynthesis and crop yield are improved by accelerating recovery from photoprotection. Sci. (80-. ). 377, 851–854. doi: 10.1126/science.adc9831 35981033

[B18] De Wilde C. Van Houdt H. De Buck S. Angenon G. De Jaeger G. Depicker A. (2000). Plants as bioreactors for protein production: Avoiding the problem of transgene silencing. Plant Mol. Biol. 43, 347–359. doi: 10.1023/a:1006464304199 10999415

[B19] Driever S. M. Simkin A. J. Alotaibi S. Fisk S. J. Madgwick P. J. Sparks C. A.. (2017). Increased sbpase activity improves photosynthesis and grain yield in wheat grown in greenhouse conditions. Philos. Trans. R. Soc B Biol. Sci. 372, 20160384. doi: 10.1098/rstb.2016.0384 PMC5566882 28808101

[B20] Edwards G. Walker D. A. (2003). C3,C4: mechanism, and cellular and environmental regulation, of photosynthesis. (Oxford: Blackwell Scientific Publications).

[B21] Ermakova M. Lopez-Calcagno P. E. Raines C. A. Furbank R. T. von Caemmerer S. (2019). Overexpression of the rieske FeS protein of the cytochrome b 6 f complex increases C4 photosynthesis in setaria viridis. Commun. Biol. 2, 314. doi: 10.1038/s42003-019-0561-9 31453378 PMC6697696

[B22] Evron Y. Johnson E. A. Mccarty R. E. (2000). Regulation of proton flow and ATP synthesis in chloroplasts. J. Bioenerg. Biomembr. 32, 501–506. doi: 10.1023/A:1005669008974 15254385

[B23] Flügge U. I. Häusler R. E. Ludewig F. Gierth M. (2011). The role of transporters in supplying energy to plant plastids. J. Exp. Bot. 62, 2381–2392. doi: 10.1093/jxb/erq361 21511915

[B24] Foyer C. H. Bloom A. J. Queval G. Noctor G. (2009). Photorespiratory metabolism: Genes, mutants, energetics, and redox signaling. Annu. Rev. Plant Biol. 60, 455–484. doi: 10.1146/annurev.arplant.043008.091948 19575589

[B25] Fu X. Gregory L. M. Weise S. E. Walker B. J. (2022a). Integrated flux and pool size analysis in plant central metabolism reveals unique roles of glycine and serine during photorespiration. Nat. Plants. 203–222 doi: 10.1038/s41477-022-01294-9 36536013

[B26] Fu X. Smith K. Gregory L. Roze L. Walker B. (2022b). Modifying photorespiration to optimize crop performance. Understanding and improving crop photosynthesis. Sharwood R.. (editor), Understanding and improving crop photosynthesis, Cambridge, UK: Burleigh Dodds Science Publishing, 2023. www.bdspublishing.com.

[B27] Garcia-Molina A. Leister D. (2020). Accelerated relaxation of photoprotection impairs biomass accumulation in arabidopsis. Nat. Plants 6, 9–12. doi: 10.1038/s41477-019-0572-z 31907400

[B28] Gilmour D. J. (2019). Microalgae for biofuel production. Adv Appl Microbiol. 109:1–30. doi: 10.1016/bs.aambs.2019.10.001 31677645

[B29] Hahn A. Vonck J. Mills D. J. Meier T. Kühlbrandt W. (2018). Structure, mechanism, and regulation of the chloroplast ATP synthase. Science 360, eaat4318. doi: 10.1126/science.aat4318 29748256 PMC7116070

[B30] HAY R. K. M. (1995). Harvest index: a review of its use in plant breeding and crop physiology. Ann. Appl. Biol. 126, 197–216. doi: 10.1111/j.1744-7348.1995.tb05015.x

[B31] Hill R. Bendall F. (1960). Function of the two cytochrome components in chloroplasts: A working hypothesis. Nature 186, 136–137. doi: 10.1038/186136a0

[B32] Kanazawa A. Kramer D. M. (2002). *In vivo* modulation of nonphotochemical exciton quenching (NPQ) by regulation of the chloroplast ATP synthase. Proc. Natl. Acad. Sci. U. S. A. 99, 12789–12794. doi: 10.1073/pnas.182427499 12192092 PMC130538

[B33] Kaushal C. Abdin M. Z. Kumar S. (2020). Chloroplast genome transformation of medicinal plant artemisia annua. Plant Biotechnol. J. 18, 2155–2157. doi: 10.1111/pbi.13379 32191371 PMC7589236

[B34] Kramer D. M. Cruz J. A. Kanazawa A. (2003). Balancing the central roles of the thylakoid proton gradient. Trends Plant Sci. 8, 27–32. doi: 10.1016/S1360-1385(02)00010-9 12523997

[B35] Kramer D. M. Evans J. R. (2011). The importance of energy balance in improving photosynthetic productivity. Plant Physiol. 155, 70–78. doi: 10.1104/pp.110.166652 21078862 PMC3075755

[B36] Kramer D. M. Wise R. R. Frederick J. R. Alm D. M. Hesketh J. D. Ort D. R.. (1990). Regulation of coupling factor in field-grown sunflower: A redox model relating coupling factor activity to the activities of other thioredoxin-dependent chloroplast enzymes. Photosynth. Res. 26, 213–222. doi: 10.1007/BF00033134 24420586

[B37] Kromdijk J. Głowacka K. Leonelli L. Gabilly S. T. Iwai M. Niyogi K. K.. (2016). Improving photosynthesis and crop productivity by accelerating recovery from photoprotection. Sci. (80-. ). 354, 857–861. doi: 10.1126/science.aai8878 27856901

[B38] Li X.-P. Muller-Moule P. Gilmore A. M. Niyogi K. K. (2002). PsbS-dependent enhancement of feedback de-excitation protects photosystem II from photoinhibition. Proc. Natl. Acad. Sci. U. S. A. 99, 15222–15227. doi: 10.1073/pnas.232447699 12417767 PMC137571

[B39] Lin M. T. Occhialini A. Andralojc P. J. Devonshire J. Hines K. M. Parry M. A. J.. (2014). β-carboxysomal proteins assemble into highly organized structures in nicotiana chloroplasts. Plant J. 79, 1–12. doi: 10.1111/tpj.12536 24810513 PMC4080790

[B40] Livingston A. K. Cruz J. A. Kohzuma K. Dhingra A. Kramer D. M. (2010). An arabidopsis mutant with high cyclic electron flow around photosystem I (hcef) involving the NADPH dehydrogenase complex. Plant Cell 22, 221–233. doi: 10.1105/tpc.109.071084 20081115 PMC2828696

[B41] Long S. P. Zhu X.-G. Naidu S. L. Ort D. R. (2006). Can improvement in photosynthesis increase crop yields? Plant Cell Environ. 29, 315–330. doi: 10.1111/j.1365-3040.2005.01493.x 17080588

[B42] Ma F. Jazmin L. J. Young J. D. Allen D. K. (2014). Isotopically nonstationary 13C flux analysis of changes in arabidopsis thaliana leaf metabolism due to high light acclimation. Proc. Natl. Acad. Sci. U. S. A. 111, 16967–16972. doi: 10.1073/pnas.1319485111 25368168 PMC4250135

[B43] Miller T. E. Beneyton T. Schwander T. Diehl C. Girault M. McLean R.. (2020). Light-powered CO2 fixation in a chloroplast mimic with natural and synthetic parts. Sci. (80-. ). 368, 649–654. doi: 10.1126/science.aaz6802 PMC7610767 32381722

[B44] Mitchell P. (1975). The protonmotive q cycle: a general formulation. FEBS Lett. 59, 137–139. doi: 10.1016/0014-5793(75)80359-0 1227927

[B45] Mitchell P. (2011). Chemiosmotic coupling in oxidative and photosynthetic phosphorylation. 1966. Biochim. Biophys. Acta 1807, 1507–1538. doi: 10.1016/j.bbabio.2011.09.018 22082452

[B46] Miyagawa Y. Tamoi M. Shigeoka S. (2001). Overexpression of a cyanobacterial. Nat. Biotechnol. 19, 965–969. doi: 10.1038/nbt1001-965 11581664

[B47] Müller P. Li X. P. Niyogi K. K. (2001). Non-photochemical quenching. a response to excess light energy. Plant Physiol. 125, 1558–1566. doi: 10.1104/pp.125.4.1558 11299337 PMC1539381

[B48] Murchie E. H. Niyogi K. K. (2011). Manipulation of photoprotection to improve plant photosynthesis. Plant Physiol. 155, 86–92. doi: 10.1104/pp.110.168831 21084435 PMC3075776

[B49] Nicholls D. Ferguson S. (2002). Bioenergetics. 3rd ed (London: Academic Press Inc).

[B50] Nimmo I. C. Barbrook A. C. Lassadi I. Chen J. E. Geisler K. Smith A. G.. (2019). Genetic transformation of the dinoflagellate chloroplast. Elife 8, 1–15. doi: 10.7554/eLife.45292 PMC6639071 31317866

[B51] Niyogi K. K. Grossman A. R. Björkman O. (1998). Arabidopsis mutants define a central role for the xanthophyll cycle in the regulation of photosynthetic energy conversion. Plant Cell 10, 1121–1134. doi: 10.1105/tpc.10.7.1121 9668132 PMC144052

[B52] Noctor G. Foyer C. H. (1998). A re-evaluation of the ATP:NADPH budget during C3 photosynthesis: A contribution from nitrate assimilation and its associated respiratory activity? J. Exp. Bot. 49, 1895–1908. doi: 10.1093/jxb/49.329.1895

[B53] Ogren W. L. (1984). Photorespiration: Pathways, regulation, and modification. Annu. Rev. Plant Physiol. 35, 415–442. doi: 10.1146/annurev.pp.35.060184.002215

[B54] Ohlrogge J. Browse J. (1995). Lipid biosynthesis. Plant Cell 7, 957–970. doi: 10.1105/tpc.7.7.957 7640528 PMC160893

[B55] O’Neill E. C. Kelly S. (2017). Engineering biosynthesis of high-value compounds in photosynthetic organisms. Crit. Rev. Biotechnol. 37, 779–802. doi: 10.1080/07388551.2016.1237467 27701897

[B56] Ort D. R. Merchant S. S. Alric J. Barkan A. Blankenship R. E. Bock R.. (2015). Redesigning photosynthesis to sustainably meet global food and bioenergy demand. Proc. Natl. Acad. Sci. U. S. A. 112, 8529–8536. doi: 10.1073/pnas.1424031112 26124102 PMC4507207

[B57] Paul M. J. (2021). What are the regulatory targets for intervention in assimilate partitioning to improve crop yield and resilience? J. Plant Physiol. 266, 153537. doi: 10.1016/j.jplph.2021.153537 34619557

[B58] Peterhansel C. Horst I. Niessen M. Blume C. Kebeish R. Kürkcüoglu S.. (2010). Photorespiration. Arab. B. 8, e0130. doi: 10.1199/tab.0130 PMC3244903 22303256

[B59] Reiland S. Messerli G. Baerenfaller K. Gerrits B. Endler A. Grossmann J.. (2009). Large-Scale arabidopsis phosphoproteome profiling reveals novel chloroplast kinase substrates and phosphorylation networks. Plant Physiol. 150, 889–903. doi: 10.1104/pp.109.138677 19376835 PMC2689975

[B60] Rott M. Martins N. F. Thiele W. Lein W. Bock R. Kramer D. M.. (2011). ATP synthase repression in tobacco restricts photosynthetic electron transport, CO2 assimilation, and plant growth by overacidification of the thylakoid lumen. Plant Cell 23, 304–321. doi: 10.1105/tpc.110.079111 21278125 PMC3051256

[B61] Ruf S. Kroop X. Bock R. (2021). Chloroplast transformation in arabidopsis. Curr. Protoc. 1, 1–24. doi: 10.1002/cpz1.103 33905600

[B62] Schwander T. Borzyskowski L. S. Von, Burgener S. Socorro Cortina N. Erb T. J. (2016). A synthetic pathway for the fixation of carbon dioxide *in vitro* . Sci. (80-. ). 354, 900–904. doi: 10.1126/science.aah5237.A PMC5892708 27856910

[B63] Seelert H. Dencher N. A. Müller D. J. (2003). Fourteen protomers compose the oligomer III of the proton-rotor in spinach chloroplast ATP synthase. J. Mol. Biol. 333, 337–344. doi: 10.1016/j.jmb.2003.08.046 14529620

[B64] Sharkey T. D. Weise S. E. (2016). The glucose 6-phosphate shunt around the Calvin-Benson cycle. J. Exp. Bot. 67, 4067–4077. doi: 10.1093/jxb/erv484 26585224

[B65] South P. F. Cavanagh A. P. Liu H. W. Ort D. R. (2019). Synthetic glycolate metabolism pathways stimulate crop growth and productivity in the field. Sci. (80-. ). 363, 0–10. doi: 10.1126/science.aat9077 PMC7745124 30606819

[B66] Strand D. D. Fisher N. Kramer D. M. (2016). “Distinct energetics and regulatory functions of the two major cyclic electron flow pathways in chloroplasts,” in Chloroplasts: Current research and future trends. Ed. Kirchhoff H. (United Kingdom: Caister Academic Press), 89–100. doi: 10.21775/9781910190470.04

[B67] Strand D. D. Fisher N. Kramer D. M. (2017). The higher plant plastid NAD(P)H dehydrogenase-like complex (NDH) is a high efficiency proton pump that increases ATP production by cyclic electron flow. J. Biol. Chem. 292, 11850–11860. doi: 10.1074/jbc.M116.770792 28559282 PMC5512078

[B68] Strand D. D. Livingston A. K. Satoh-Cruz M. Froehlich J. E. Maurino V. G. Kramer D. M. (2015). Activation of cyclic electron flow by hydrogen peroxide *in vivo* . Proc. Natl. Acad. Sci. U. S. A. 112, 5539–5544. doi: 10.1073/pnas.1418223112 25870290 PMC4418880

[B69] Suorsa M. Järvi S. Grieco M. Nurmi M. Pietrzykowska M. Rantala M.. (2012). PROTON GRADIENT REGULATION5 is essential for proper acclimation of arabidopsis photosystem I to naturally and artificially fluctuating light conditions. Plant Cell 24, 2934–2948. doi: 10.1105/tpc.112.097162 22822205 PMC3426124

[B70] Svab Z. Maliga P. (1993). High-frequency plastid transformation in tobacco by selection for a chimeric aadA gene. Proc. Natl. Acad. Sci. U. S. A. 90, 913–917. doi: 10.1073/pnas.90.3.913 8381537 PMC45780

[B71] Verma S. Kuila A. (2020). Involvement of green technology in microalgal biodiesel production. Rev. Environ. Health 35, 173–188. doi: 10.1515/reveh-2019-0061 32134737

[B72] Walker B. J. Kramer D. M. Fisher N. Fu X. (2020). Flexibility in the energy balancing network of changing environmental conditions. Plants 9, 301. doi: 10.3390/plants9030301 32121540 PMC7154899

[B73] Wang L. M. Shen B. R. Li B. Zhang C. L. Lin M. Tong P. P.. (2020). A synthetic photorespiratory shortcut enhances photosynthesis to boost biomass and grain yield in rice. Mol. Plant 13, 1802–1815. doi: 10.1016/j.molp.2020.10.007 33075506

[B74] Wieloch T. Sharkey T. D. (2022). Compartment-specific energy requirements of photosynthetic carbon metabolism in camelina sativa leaves. Planta 255, 1–10. doi: 10.1007/s00425-022-03884-5 PMC9005430 35415783

[B75] Wu G. Ortiz-Flores G. Ortiz-Lopez A. Ort D. R. (2007). A point mutation in atpC1 raises the redox potential of the arabidopsis chloroplast ATP synthase gamma-subunit regulatory disulfide above the range of thioredoxin modulation. J. Biol. Chem. 282, 36782–36789. doi: 10.1074/jbc.M707007200 17959606

[B76] Yu S. M. Lo S. F. Ho T. H. D. (2015). Source-sink communication: Regulated by hormone, nutrient, and stress cross-signaling. Trends Plant Sci. 20, 844–857. doi: 10.1016/j.tplants.2015.10.009 26603980

[B77] Zhao L. Chang W. Xiao Y. Liu H. Liu P. (2013). Methylerythritol phosphate pathway of isoprenoid biosynthesis. Annu. Rev. Biochem. 82, 497–530. doi: 10.1146/annurev-biochem-052010-100934 23746261 PMC5031371

